# Reducing Point-of-care Blood Gas Testing in the Intensive Care Unit through Diagnostic Stewardship: A Value Improvement Project

**DOI:** 10.1097/pq9.0000000000000284

**Published:** 2020-06-24

**Authors:** Michael J. Tchou, Sally May, John Holcomb, Ethan Tanner-Edwards, Kathy Good, Matthew Frazier, Erika L. Stalets, Maya Dewan

**Affiliations:** From the * Department of Pediatrics, University of Colorado School of Medicine, Aurora, Colo.; † Section of Hospital Medicine, Children’s Hospital of Colorado, Aurora, Colo.; ‡ Department of Radiology, Cincinnati Children’s Hospital Medical Center, Cincinnati, Ohio; § James M. Anderson Center for Health Systems Excellence, Cincinnati Children’s Hospital Medical Center, Cincinnati, Ohio; ¶ Clinical Laboratory Administration, Cincinnati Children’s Hospital Medical Center, Cincinnati, Ohio; ∥ Department of Pediatrics, University of Cincinnati, College of Medicine, Cincinnati, Ohio; * *Division of Critical Care Medicine, Cincinnati Children’s Hospital Medical Center, Cincinnati, Ohio.

## Abstract

**Introduction:**

Overutilization of point-of-care (POC) testing may reduce the overall value of care due to high-cost cartridges, need for staff training, and quality assurance requirements.

**Methods:**

The Diagnostic Stewardship group at Cincinnati Children’s Hospital Medical Center assembled a multidisciplinary team to reduce the use of POC blood gas testing by 20% in the pediatric intensive care unit (PICU). Key drivers of test overutilization included poor knowledge of cost, concern with testing turnaround time, and a lack of a standard definition of when a POC test was appropriate. We calculated weekly the outcome measure of POC blood gas tests per PICU patient-day and a balancing measure of blood gas result turnaround time using data extracted from the electronic medical record. Interventions focused on staff education, the establishment of a standard practice guideline for the use of POC testing, and improving turnaround time for laboratory blood gas testing.

**Results:**

Over the baseline period starting July 2016, a median of 0.94 POC blood gas tests per PICU patient-day was ordered. After initial staff training, the rate was reduced to 0.60 tests per PICU patient-day and further reduced to 0.41 tests per PICU patient-day after a formal policy change was adopted. We have sustained this rate for 15 months through June 2018. Institutional direct cost savings were estimated to be $19,000 per year.

**Conclusions:**

Our improvement initiative was associated with a significant and rapid reduction in the use of POC testing in the PICU. Interventions focused on cost awareness, and a formal guideline helped establish a consensus around appropriate utilization.

## Abstract 2

Supplemental Digital Content is available in the text.

## INTRODUCTION

The overall cost of pediatric care in the United States has been steadily increasing^1,2^ and is now on par with the total health spending for several developed countries.^3^ This trend has brought into focus the need to consider the value of the services we provide across the spectrum of pediatric care environments. In particular, overutilization of healthcare resources such as laboratory testing is a frequent source of wasted healthcare spending.^4,5^

Rapid point-of-care (POC) testing is an expanding area of diagnostic testing in pediatrics.^6,7^ POC blood gas testing delivers rapid and accurate results, allowing assessment of metabolic and respiratory status in critical situations and prompt initiation of treatments.^8,9^ This has led to POC blood gas tests becoming more frequent or even standard options in several care settings, including the pediatric intensive care unit (PICU) and the emergency department.^6^ However, over-reliance on POC testing may both reduce value and increase the waste of hospital resources. First, POC testing may be more expensive than the main or central laboratory (CL) testing.^10^ Second, POC testing may be more staff intensive or may divert staff from other value-added work. Front-line staff such as nurses and respiratory therapists often perform POC testing, which involves loading a sample into the analyzer and waiting for results. In high-intensity environments like the PICU, the frequent use of POC testing adds cumulative time and task burden to these highly skilled staff that could be better utilized elsewhere.^11^ Third, utilizing POC testing may reduce the efficiency of in-laboratory testing by diverting volume away from the diagnostic laboratory where high fixed cost machines benefit from high volumes of use. Finally, although POC testing generally provides results more quickly, allowing earlier treatment, there have been mixed results on any significant changes to meaningful outcomes for patients where POC testing is used.^8,12^ For these reasons, a focused value improvement project targeting POC blood gas testing in the PICU may help improve overall value in this environment.

Our overall goal for this project was to improve the value of blood gas testing in the PICU by reducing the use of high-cost POC testing in situations where low-cost, timely CL testing with a stat turnaround time would lead to the same patient care outcomes. Our specific aim was to reduce the use of POC blood gas testing by 20% in the PICU over 6 months and demonstrate sustainability.

## METHODS

### Context

Cincinnati Children’s Hospital Medical Center is a large, quaternary care pediatric center with > 700 beds. The health system has over 33,000 annual admissions, with over 2,500 annual admissions to a 35-bed PICU.

As part of a larger utilization management effort, we established a diagnostic stewardship committee in 2015 to reduce the overuse of unnecessary laboratory testing and diagnostic imaging. The committee is composed of multidisciplinary representatives from the Department of Pathology and Laboratory Medicine and the Department of Radiology, and physicians from the Department of Pediatrics, as well as data analyst support and quality improvement coaching from the James M. Anderson Center for Health Systems Excellence.

### Planning the Intervention

The diagnostic stewardship team used the Institute for Healthcare Improvement Model for Improvement as the framework for this value improvement project.^13^ We began by assembling a multidisciplinary project team that included a subset of the diagnostic stewardship team and included a pediatric hospitalist, a pediatric critical care physician, a data analyst, a quality improvement coach, laboratory director, and hospital administrator. The team assembled and investigated a baseline dataset of current blood gas utilization and determined what proportion and in what clinical circumstances they were used in the PICU. A review of the financial impacts of testing indicated that the direct costs of POC testing were twice the costs of in-laboratory testing, and charges were significantly higher for POC testing. Cost estimates of staff time to perform testing were similar between POC testing and in-laboratory testing. To better understand the patient-level impact of the longer time to results for in-laboratory testing, the improvement team also analyzed the turnaround time for blood gas testing sent to the laboratory from the PICU.

**Fig. 1.**
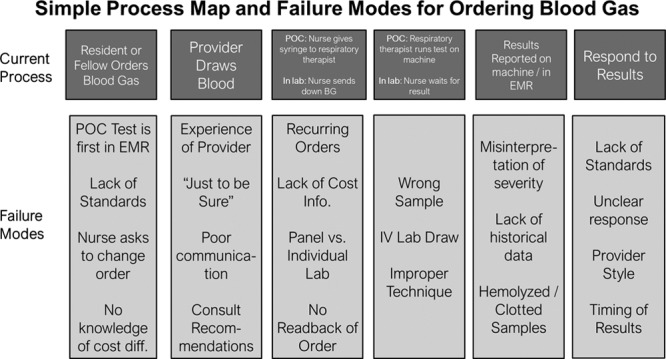
Simple process map and failure mode and effects analysis. A description of the current process of blood gas testing based on stakeholder interviews at the beginning of the improvement project and an analysis of potential failure modes at each step.

**Fig. 2.**
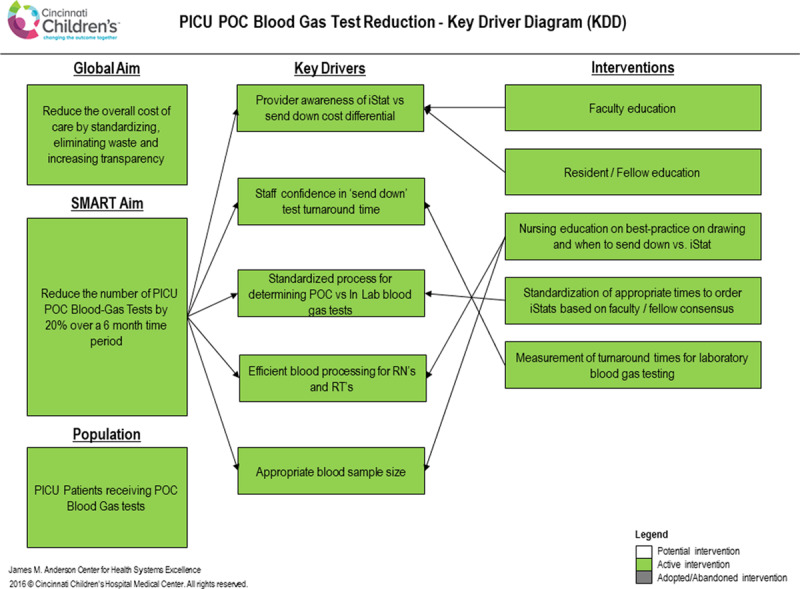
Key driver diagram. Key driver diagram indicating the theory of improvement based on stakeholder interviews and process mapping. Grayed-out key drivers were initially identified, but no intervention was performed to target these drivers directly. KDD, key driver diagram; RN, registered nurse; RT, respiratory therapist.

The improvement team interviewed front-line providers in the PICU, including attending physicians, fellows, advanced practice providers, residents, respiratory therapists, and nurses. The team also shadowed the rounding process in the PICU to better understand the process of blood gas testing and any clinical factors affecting this process. In particular, we discussed with these staff members (1) when a blood gas result was needed quickly (ie, within 5−10 minutes) and when a blood gas result within 1 hour was acceptable and (2) any knowledge the staff had about any cost differential between a POC blood gas and an in-laboratory blood gas. Through this process of stakeholder interviews, the improvement team developed a process map and failure mode and effects analysis (Fig. 1) to identify areas where interventions may be most effective. In particular, the team identified the clinical situations where testing was recurring or occurred predictably as the best opportunities for replacing POC testing with in-laboratory testing. This initial work guided the improvement team’s creation of a key driver diagram (Fig. 2). Key drivers identified by this process included (1) knowledge of the cost differential between POC testing and in-laboratory testing; (2) staff confidence that send-down testing would consistently provide an accurate result within 1 hour; (3) a standardized process for determining when staff should use POC testing and when they should not; and (4) standardized and efficient processes for central laboratory testing that minimized errors and rejected blood specimens.

We developed baseline measures and our target population for interventions based on interviews and conversations within the diagnostic stewardship team. Given the need for rapid decision-making and an established protocolized laboratory schedule for patients on extracorporeal membranous oxygenation (ECMO) and continuous renal replacement therapy (CRRT), we excluded these patients from our target population.

**Fig. 3.**
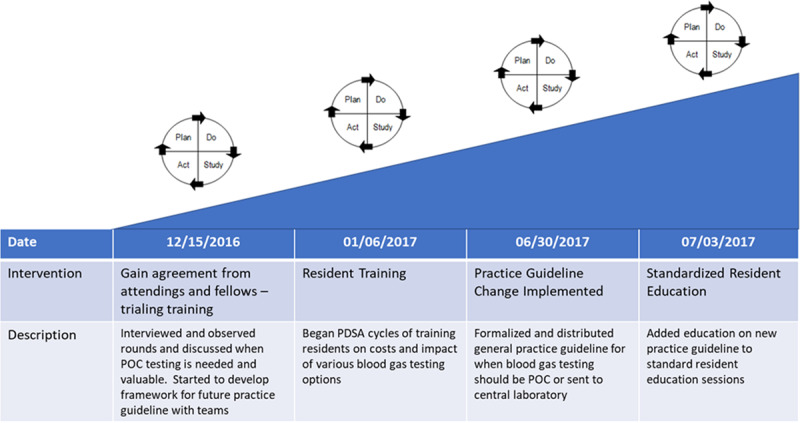
PDSA ramp of interventions. Descriptions of interventions and the timing of when they occurred for the improvement project. PDSA, plan-do-study-act.

We performed a value improvement study^14^ to determine the effects of our interventions (Fig. 3) on the rate of POC blood gas testing. This improvement project was not considered human subject research. Therefore, review and approval by the Cincinnati Children’s Hospital Medical Center institutional review board were not required.

### Improvement Activities

#### Gained Agreement from Attendings and Fellows

Initial conversations with attendings and fellows in the PICU occurred at the division meeting and achieved consensus in supporting decreased utilization of POC testing. Initial work focused on reminding the attendings and fellows as leaders of the clinical care team of our objectives for this project on an ongoing basis. In-person check-ins or email reminders by a member of the improvement team at the start of attending and fellows’ rotations reinforced the training.

#### Resident Training

One month after our initial training with attendings and fellows, we began in-person training of residents on the first day of their rotation in the PICU. These training sessions were short (≈5 minutes), informal didactics, and initially focused on increasing awareness of the cost and charge differentials of different forms of blood gas testing and the agreed-upon focus on using in-laboratory testing for low-risk, predictable clinical situations. This training was later standardized and added to the existing PICU orientation for residents in July 2017.

#### Development of Implementation of Clear Practice Guidelines for Testing

After evaluating our initial improvement work and education, the improvement team and clinical staff reached a consensus to formalize the practice change for POC blood gas testing in the PICU. An initial draft of the practice change was created and revised with feedback from PICU attendings and the PICU medical director. A final practice guideline was then presented at a PICU division meeting and circulated to staff.

#### Development of System to Triage Problems with Turnaround Time

Due to concerns about the impacts of turnaround time on patient care from in-laboratory blood gas testing, turnaround time was monitored and reviewed at improvement team meetings after the start of interventions. We reviewed weekly turnaround time to ensure that it was not increasing with increased volumes of blood gases being sent to the laboratory from the PICU. Additionally, specific cases of prolonged turnaround time were reviewed to understand system failures. This process continued until September 2017, after which the data were reviewed as needed based on any concerns from the laboratory staff or bedside clinicians.

### Study of the Interventions

Baseline data were gathered from July 2016 to November 2016 utilizing electronic medical record (EMR) data extraction of blood gas testing. To understand the impact of our interventions on rates of testing, we reviewed data every 2 weeks at improvement team meetings.

### Measures

Our primary measure was the weekly rate of POC blood gas tests per PICU patient-day. We defined *POC blood gas tests* as any POC blood gas test performed while a patient was admitted to the PICU. We excluded any POC blood gas test drawn on the calendar-day when the care of that patient included ECMO or CRRT. To account for variation in the census of patients in the PICU for any given week, we divided the number of tests by the number of PICU patient-days that week. Any calendar-day that a patient was in the PICU counted in the *PICU patient-days* denominator. All patient-days during which a patient’s care included ECMO or CRRT were excluded from our measure. After study completion, we measured total ventilator-days per month as a second measure of patient acuity to ensure no changes to acuity coincided with changes in testing rates.

To measure the impact of increasing in-laboratory blood gas utilization, we measured the average weekly turnaround time for blood gas processing once the sample arrived in the CL as our balancing measure. This time did not include the time from blood draw to delivery to the laboratory because it was not as reliably recorded. Specifically, the laboratory turnaround time for a blood gas was defined as the time between blood specimen arrival in the laboratory and the time the results were available in the EMR. The average turnaround time was calculated weekly.

To understand the impact of our interventions on overall rates of testing, the improvement team followed weekly rates of total blood gas testing, defined as the sum of total POC blood gas tests as defined above combined with CL blood gas tests divided by the total number of PICU patient-days.

To measure the financial impact of the project, we calculated direct supply costs using laboratory financial and procurement systems data and interviews with laboratory management team members. Direct supply cost savings were estimated to be $5.20 per test when using CL testing instead of POC testing. Charge estimates were obtained directly from financial systems data.

### Analysis

Measures were analyzed using statistical process control charts and run charts, and standard rules to determine special cause variation were employed.^15^ We charted POC blood gas tests per PICU patient-day on a u-chart. Turnaround time and total blood gas testing were measured weekly and tracked on run charts. Total potential cost savings were calculated by comparing preintervention rates of POC blood gas testing to postintervention rates and calculating the differential savings from converting these tests to in-laboratory blood gas testing.

## RESULTS

**Fig. 4.**
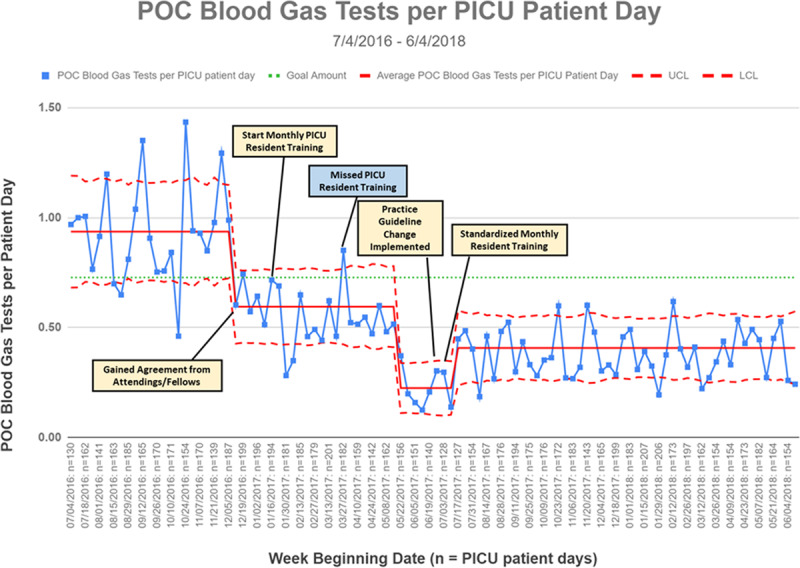
Primary measure. Statistical process control u-chart demonstrating changes to the improvement project’s primary measure of POC blood gas tests per PICU patient-day and interventions annotated in boxes. LCL, lower control limit; UCL, upper control limit.

Baseline data from July to November 2016 showed that we averaged 0.94 POC blood gas tests per PICU patient-day (Fig. 4). There was substantial weekly variation in rates of testing, ranging from 0.46 to 1.44 POC blood gas tests per PICU patient-day, and PICU census varied between 130 and 185 patient-days per week during this time. After initiating provider training, mean testing rates decreased from 0.94 to 0.60 POC tests per PICU patient-day. With further training, we observed a brief reduction to 0.23 POC blood gas tests per PICU patient-day. After a formalized practice guideline was created and implemented and training was incorporated into existing resident orientation structures, testing rates stabilized at 0.41 tests per patient-day. Variation in testing rates decreased after implementing systematic training and disseminating official practice changes, indicated by narrower control limits on our statistical process control chart. We sustained lower testing rates and lower variation in testing rates for over a year.

**Fig. 5.**
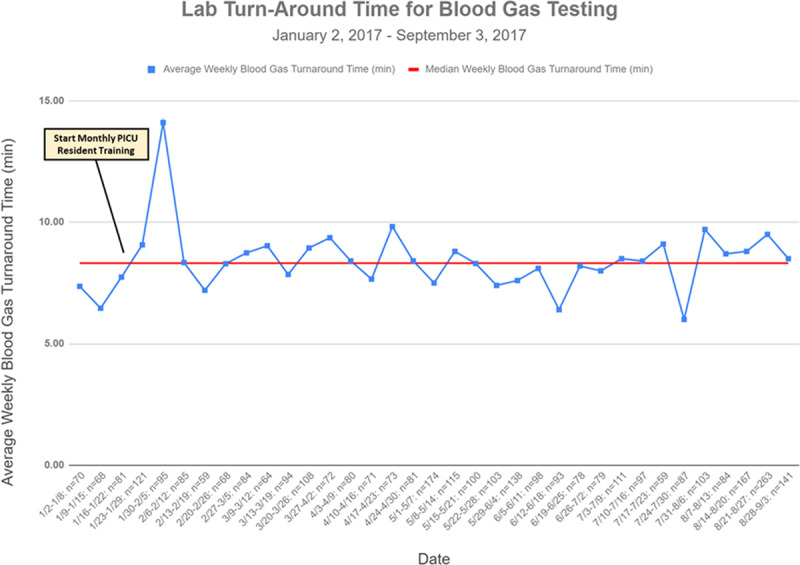
Turnaround times process measure. A run chart measuring laboratory turnaround time as a balancing measure for our primary intervention.

In-laboratory turnaround time after the start of our interventions averaged 8.3 minutes with only 1 weekly average turnaround time >10 minutes (Fig. 5). Variation in turnaround time weekly was not clinically significant, and no special cause variation was identified through the end of formal monitoring in September 2017. No significant trends of changes in overall ventilator-days per month were observed during the study period (see **Supplemental Digital Content** at http://links.lww.com/PQ9/A181).

**Fig. 6.**
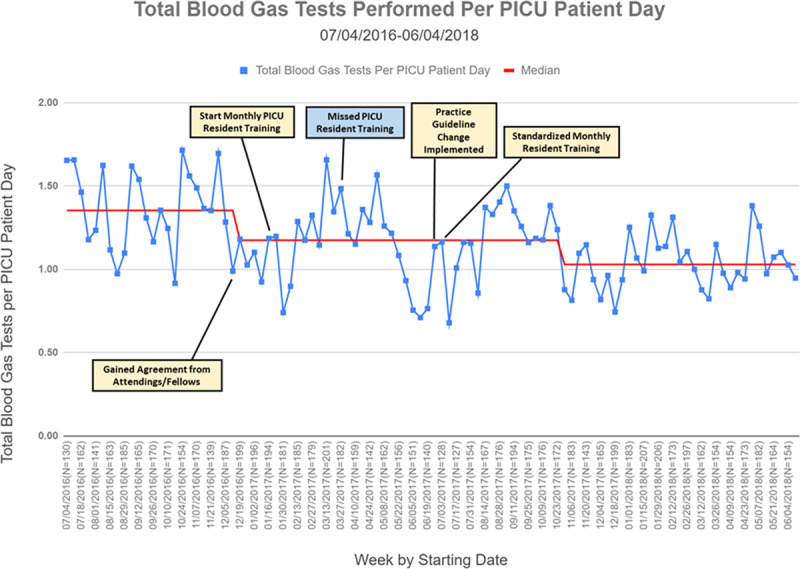
Total blood gas utilization rates. A run chart describing the rates of total blood gas utilization in the PICU during the study period.

The total blood gas testing rate in the baseline period was 1.35 total blood gas tests per PICU patient-day (Fig. 6). After the start of interventions, a special cause variation occurred, and testing rates decreased to 1.17 total blood gas tests per PICU patient-day. Several nonsustained special cause variations occurred during the time of interventions, and a final special cause variation occurred after standardization of training and guidelines leading to a decrease in testing rate to 1.03 total blood gas tests per PICU patient-day.

Based on a sustained postintervention testing rate of 0.41 POC blood gas tests per PICU patient-day, we calculated the total estimated direct supply cost savings to be $19,068 per year, with estimated reduced annual potential patient charges of ≈$1.2 million.

## DISCUSSION

Using improvement science methods, we demonstrated a rapid and sustained 56% reduction in the use of POC blood gas testing in the PICU and a decrease in the weekly variability in utilization rates. This reduction resulted in a net decrease in direct costs to the institution for blood gas testing in the PICU and a net decrease in overall blood gas testing despite increased utilization of the CL. Overall, blood gas testing decreased most after training, and guidelines were standardized. Our balancing measure, laboratory turnaround time, did not change either during or after interventions, indicating they remained constant despite an increase in tests sent to the CL. Training and increased awareness about the financial impacts of different types of testing correlated with initial reductions in testing, and formal practice changes and standardized education led to further reductions. This reduced rate was sustained for over a year and supported the idea that the practice changes were acceptable and feasible for staff in the PICU. Although no formal measurement of acceptability occurred in this project, there is evidence that a strategy of substituting a low-value practice with a higher-value practice may be more acceptable to and create less negative reactions from providers compared with interventions that ask providers to stop a specific behavior altogether.^16^ The framing of our intervention in this manner may have helped with sustainment. Also, more standardization and definition of the specific clinical scenarios where POC should be used may have contributed to a decrease in the variation in rates of utilization throughout the postintervention year. Importantly, although some previous value improvement work has used EMRs to provide decision support, our improvement work was sustained without a specific EMR intervention and the resulting challenges of alert fatigue that sometimes accompany these interventions.^17^

Studies on the value and cost of POC testing in high-acuity settings are mixed. Some work indicates that going completely to POC testing may save money in specific high-risk or time-sensitive clinical scenarios such as stabilization before transport^18^ and in the emergency department.^19^ However, the financial evaluation is complicated with inconsistent results about when POC testing may improve overall value.^10,12,20,21^ These financial evaluations are complex^10,22^; however, one important consideration is the opportunity costs of testing with POC compared with in-laboratory testing. When an onsite laboratory can provide STAT results with minimal turnaround time, some have argued that the benefits of POC testing may be minimal except in the most urgent of situations.^23^ One reason for this is that it is more efficient to maximize the use of an existing machine with a high fixed cost over POC testing where testing cartridges have a relatively higher variable cost that changes with utilization volume.^6,10^ When there is the capacity to process blood gases rapidly within the existing diagnostic laboratory easily, the CL machine is used most efficiently. Additionally, time is also freed up for other staff to provide value-added work, and the use of the CL minimizes the chances of preanalytic errors by clinical staff that may occur with POC test samples.^7,24,25^

Because this context and understanding of systems-level processes are key for determining value, the use of a quality improvement mindset for approaching value improvement is important.^26^ Process mapping, stakeholder interviews, and key driver diagrams can help a diagnostic stewardship team understand the systems and context that may be important to a specific care environment. In another situation such as a more remote hospital unit where blood gas testing would involve a courier to a laboratory, the financial evaluation may favor POC testing over in-laboratory testing due to increased variable costs for transport and time to complete the delivery. In the end, carefully considering the context of the hospital system is key to understanding what may be the most high-value strategy for specific categories of testing, and careful evaluation of context and systems is important for future diagnostic stewardship and value improvement work.

Overall, as reimbursement systems for hospitalization shift to more value-based payment mechanisms, the PICU may be an important opportunity for hospital systems to consider when evaluating how to improve the overall value of care. In most hospitals, the PICU is a high-utilization area where we have reported that high-value care education does not occur as frequently.^27^ However, previous projects have demonstrated success in reducing utilization in intensive care settings through standardization approaches similar to ours.^28^ Establishing a culture of value, and careful evaluations of testing necessity and choice of testing modality, are important parts of improving the value of care, even in the critical care setting.^29^

## LIMITATIONS

Our work should be evaluated with consideration of several specific limitations. We observed no negative downstream morbidity or mortality related to utilizing in-laboratory testing instead of POC testing, but due to the volume of PICU patients and the small number of negative outcomes, it is possible that clinical scenarios where there was negative consequence occurred. Importantly, no specific serious safety events or root cause analyses occurred during the improvement period that identified cases with delayed turnaround time. In all cases of delayed turnaround time reviewed by the improvement team, no significant events occurred. Our turnaround time balancing measure did not include the time from obtaining the blood sample to the time it arrived in the laboratory. Although we did not find any adverse effects related to turnaround time, measuring this preanalytic period may be important in certain clinical contexts or with certain clinical workflows if this work is adapted to other settings. The financial impact is difficult to measure due to complex hospital systems with a mix of direct, indirect, fixed, and variable costs. However, our study results are focused on direct, measurable costs. To further understand the impacts and trade-offs of POC blood gas testing and in-laboratory blood gas testing in the PICU, detailed cost-effectiveness analysis may be necessary.

## CONCLUSIONS

Our value improvement project successfully reduced the use of POC testing in a high-volume PICU and sustained those changes in test ordering practice for over a year. Although POC testing can provide accurate, rapid results in situations where this information is time-critical, overuse of POC testing may decrease the value of care and increase resource waste in certain situations. Careful consideration of the overall processes and care systems can help hospitals identify areas where decreased utilization of POC testing may reduce costs and improve the value of care.

## DISCLOSURE

The authors have no financial interest to declare in relation to the content of this article.

## ACKNOWLEDGMENTS

The authors acknowledge the Cincinnati Children’s Hospital Medical Center Diagnostic Stewardship Team and the James M. Anderson Center for Health Systems Excellence for supporting this project, and the PICU staff for their enthusiastic participation. Specifically, the authors offer thanks to Joshua McDonald for assistance in the automation of our primary measure, and the Cincinnati Children’s Hospital Pediatric Hospital Medicine Fellowship for their support of Dr. Tchou’s improvement training.

For a full list of members of the Cincinnati Children’s Hospital Medical Center Diagnostic Stewardship Committee who are not in the authorship team, please visit http://links.lww.com/PQ9/A203.^29^

## Supplementary Material


