# Supplementary figures and images for: Reducing Point-of-care Blood Gas Testing in the Intensive Care Unit through Diagnostic Stewardship: A Value Improvement Project
Source: Pediatr Qual Saf. 2020 Jun 24;5(4):e284. doi: 10.1097/pq9.0000000000000284 (PMC7339248; doi:10.1097/pq9.0000000000000284)

**Supplement 1: Total Ventilator Days by Month Throughout the Improvement Project**

**
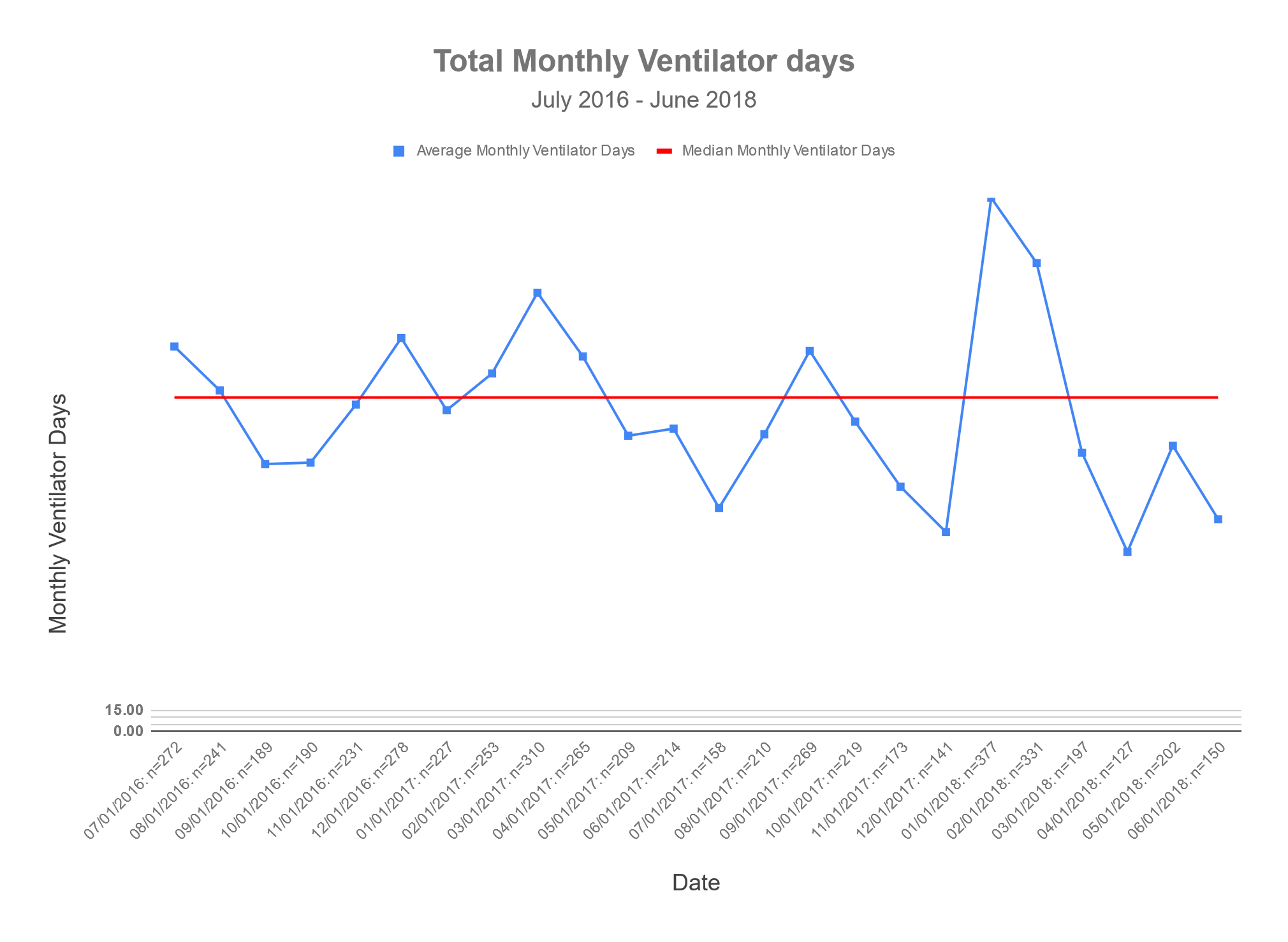
**

Supplement: Supplementary file 1 [file pqs-5-e284-s001.docx]
