# Supplementary material for: Reducing Point-of-care Blood Gas Testing in the Intensive Care Unit through Diagnostic Stewardship: A Value Improvement Project
Source: Pediatr Qual Saf. 2020 Jun 24;5(4):e284. doi: 10.1097/pq9.0000000000000284 (PMC7339248; doi:10.1097/pq9.0000000000000284)
Supplement: Supplementary file 2 [file pqs-5-e284-s002.docx]

**List of Members of the Cincinnati Children’s Hospital Medical Center Diagnostic Stewardship Committee who are not in the authorship team**

Paul Steele – Medical Director, Clinical Labs

Susan Lee – Business Director, Clinical Labs

Andy Spooner – CMIO, Information Services

Nick DeBlasio – Assistant Professor, General Pediatrics

Brandy Seger – Lead Quality Outcomes Manager, Anderson Center

Danny Mallon – Assistant Professor, Gastroenterology

Jeff Anderson – Associate Professor, Heart Institute

Josh Courter – Clinical Specialist, Pharmacy

Brian Dawson – Professor, Human Genetics

Tierney Morrison – Chief Resident, Pediatric Residency Training Program

Austin Ostermeier – Chief Resident, Pediatric Residency Training Program

Allison Divanovic – Associate Professor, Heart Institute

Lisa Ulland – Business Director II, Radiology

Laura Lewin – Business Director II, Heart Institute

Stephanie Kinney – Assistant Professor, Pathology

Katie Wusik Healy – Genetic Counselor III, Human Genetics

Kathleen Collins – Genetic Counselor III, Human Genetics
